# Diagnosis of *Trichomonous vaginalis* by microscopy, latex agglutination, diamond’s media, and PCR in symptomatic women, Khartoum, Sudan

**DOI:** 10.1186/1746-1596-9-49

**Published:** 2014-03-06

**Authors:** Amir M Saleh, Hamid S Abdalla, Abdelsalam B Satti, Suad M Babiker, Gasim I Gasim, Ishag Adam

**Affiliations:** 1 Faculty of Medical Laboratory Sciences, University of Omdurman, Omdurman, Sudan; 2 College of Medicine, Qassim University, Buraydah, Qassim, Saudi Arabia; 3 Faculty of Medicine, University of Khartoum, Khartoum, Sudan

**Keywords:** Latex agglutination, Diamond’s media, PCR, *Trichomonas vaginalis*, Sudan

## Abstract

**Background:**

Trichomoniasis is the most common sexually transmitted disease. However, limited data are available on an effective technique for the diagnosis of *Trichomonas vaginalis*.

**Methods:**

A cross-sectional study was conducted to evaluate the accuracy of wet mount microscopy, latex agglutination, Diamond’s media, and polymerase chain reaction (PCR) for detection of *T. vaginalis* among symptomatic women who attended the gynecological clinic at Khartoum, Sudan.

**Results:**

Of the 297 women studied, 252 (84.8%) were positive for *T. vaginalis* by wet mount microscopy, 257 (86.5%) by latex agglutination, 253 (85.2%) by Diamond’s media, and 253 (85.2%) by PCR. The sensitivity and specificity of wet mount microscopy were 99.2% and 97.7%, respectively, compared with PCR. The sensitivity and specificity of latex agglutination and Diamond’s media were 99.6% and 88.6%, and 100.0% and 86.4%, respectively, compared with PCR.

**Conclusions:**

In this study, wet mount microscopy, latex agglutination, and Diamond’s media were found to be highly sensitive and specific. However, the availability and cost effectiveness might limit the use of Diamond’s media and PCR in routine practice.

**Virtual slides:**

The virtual slide(s) for this article can be found here: http://www.diagnosticpathology.diagnomx.eu/vs/7859723851211496.

## Background

*Trichomonas vaginalis* is a flagellated eukaryotic organism, which has caused a worldwide health problem, with an estimated 170 million people infected annually with *T. vaginalis*[1,2]. Infection with *Trichomonas* is associated with a significant risk of morbidity in women, including pelvic inflammatory disease, adverse pregnancy outcomes (e.g., preterm labor and delivery), cervical dysplasia, infertility, increased risk of postoperative infection, and human immunodeficiency virus acquisition and transmission [3-5].

Therefore, accurate diagnosis of *T. vaginalis* infection is an important issue. *T. vaginalis* infection cannot be accurately diagnosed based on the clinical picture because clinical symptoms of trichomoniasis may be similar to those of other sexually transmitted diseases [6]. Different diagnostic methods that are usually used (wet mount method) for routine field diagnosis of *T. vaginalis* have low sensitivity [7,8]. Other methods for diagnosis of *T. vaginalis,* such as culture and polymerase chain reaction (PCR), are time consuming and not available in all field laboratories [8]. Therefore, a simple and rapid diagnostic test with acceptable sensitivity and specificity is required. The latex agglutination test has a high sensitivity and specificity [8,9]. However, there are few recently published data on the performance of different diagnostic methods for *T. vaginalis* and no data are available in Sudan.

The current study was conducted to evaluate the performance of wet mount microscopy, latex agglutination, Diamond’s media, and PCR for detection of *T. vaginalis* among symptomatic women who attended the gynecological clinic in Khartoum, Sudan. Findings from this study will be of great importance for care givers and researchers, and it will add to the diagnostic tools for different diseases among pregnant and non-pregnant Sudanese women [10-12].

## Methods

A cross-sectional study was conducted during April to August 2012 at Khartoum, Sudan. After signing an informed consent and recording the socio-demographic characteristics and symptomatic history (vaginal discharge, itching, dysuria, dyspareunia, and foul odor) women who attended the gynecological clinic in Khartoum were approached to participate in the study.

Cotton-tipped applicators were used to obtain vaginal fluid swabs from each woman using a vaginal speculum. At least three swabs were collected in a sterile test tube that contained 0.2 ml of 5% glucose saline. This tube was maintained at body temperature for wet mount examination, which was performed within 10 minutes of collection of the specimen. The applicator was gently agitated in the saline, and a wet mount on a clean slide was prepared and observed under a microscope for motile trichomonads (400×) to confirm flagellar movement, morphological features, and the number of *T. vaginalis.*

The wet preparation was then inoculated into Diamond’s media, the latex agglutination test was applied, and *T vaginalis* DNA was detected by real-time PCR. An Amplicor swab (Roche Molecular) was used to collect specimens for PCR amplification, while Dacron-tipped swabs were used to collect materials for microscopic examination, the latex agglutination test, and culturing of the organism.

### Diamond’s media

A total of 30 g of Diamond’s media was suspended in 900 ml of purified filtered water. This was then heated with frequent agitation, boiled for 1 minute, and then sterilized at 121°C for 15 minutes. After cooling to 45–50°C, 100 ml of horse serum and antibiotics were added and mixed thoroughly, and were dispensed into sterile culture tubes. The prepared media was stored at 2–8°C and protected from direct light, and dehydrated powder was stored in a dry place in tightly-sealed containers at 2–25°C. Prior to inoculation, the media was brought up to 35–37°C in an aerobic incubator for approximately 1–2 hours. Specimens were examined for *T. vaginalis* prior to inoculation into culture media. Tubes were incubated aerobically in a slanted position (45° angle) at 35–37°C. Cultures were examined by wet mount for motile trophozoites after 24 or 48 hours. Centrifugation of the culture was used to detect low-level growth, and then examined microscopically daily by adding one drop on a slide, and covering with a cover slip. Negative cultures were incubated for at least 96 hours and examined before reporting the culture.

### Quality control of diamond’s media

An internal quality control for Diamond’s media was included by incubating one tube of Diamond’s media per batch to monitor the sterility of reagents. One tube of Diamond’s media was inoculated with a known culture of *T. vaginalis.* To check the quality of the batch of Diamond’s media, another tube of Diamond’s media was inoculated with a known culture.

### Latex agglutination test

All reagents were brought to room temperature. The test latex was shaken well immediately before use. A volume of 50 μL of the vaginal swab suspension was eluted by agitation in phosphate-buffered saline. One drop of test latex was added. Both liquids were stirred to a completely homogenous mixture that covered the whole surface of the reaction zone. The glass slide was tilted with a rotating action continuously for 3 minutes. After 3 minutes, the degree of agglutination was obtained.

The degree of agglutination was recorded as follows. Latex that had agglutinated with a lot of accumulation around the edge of the reaction zone was recoded as positive+++. Agglutinated particles that were clearly seen against a background of granular latex were recorded as positive++. Agglutination that was just able to be discerned compared with a negative control was recorded as positive+. No agglutination compared with a negative control was negative.

A negative control was used in an adjacent reaction zone in parallel with a test sample to distinguish between a weak positive and negative result. A positive control was used to monitor the performance of the test latex. Running a positive control the first time the kit was used was recommended, as well as when removed from storage.

Positive and negative controls were run every time the latex agglutination test was performed. A negative test was repeated to ensure the results.

### PCR

The target for the real-time PCR assay was a 67-base pair region of a repeated sequence of the *T. vaginalis* genome (Gene Bank Accession Number L23861). The primers and probes were designed using Primer Express (Applied Biosystems, Foster City, CA, USA). The PCR reaction contained 25 μl of 2× TaqMan1 Universal Master Mix (Applied Biosystems, manufactured by Roche, Branchburg, NJ, USA), with 5 ml each (final concentration of 900 nM) of TV forward primer (Tv3, 5′-ATTGTCGAACATTGGTCTTACCTC-3) and TV reverse primer (Tv7, 5′-TCTGTGCCGTCTTCAAGTATGC-3′), 5 ml (final concentration of 225 nM) of probe (5′FAM-TCA146 A.M. [13]. TTT CGG ATG GTC AAG CAG CCA-TAMRA-3′), and 5 μl of sample DNA. A volume of 5 μl of eluted DNA was used as a template in a final volume of 25 μl with PCR buffer (Hotstar Taq Mastermix; QIN gene), 25 μg of bovine serum albumin, 5 Mm Mg C12, and 2.0 pmol of each primer. The thermocycler used was set to provide amplification consisting of 15 minutes at 95°C followed by 50 cycles of 15 seconds at 95°C, and 1 minute at 60°C. Amplification detection and data analysis were performed with the Applied Biosystems 7500 Real Time PCR System. The PCR output from this system consisted of a cycle threshold (Ct) value, representing the amplification cycle in which the signal exceeded the background fluorescence, and indicating the parasite-specific DNA load in the sample tested.

A sample size of 295 subjects was calculated using the OpenEpi-Epidemiological calculator with 80% power and a confidence interval of 95% to detect a difference of 5% at α = 0.05, with 10% of non-respondents/incomplete data.

Ethical approval for the study was obtained from the Khartoum Hospital Ethical Board.

### Statistics

Data were analyzed using SPSS software version 19.0. Sensitivity, specificity, and positive and negative predictive values were determined as described previously [14].

## Results

**Figure 1:**
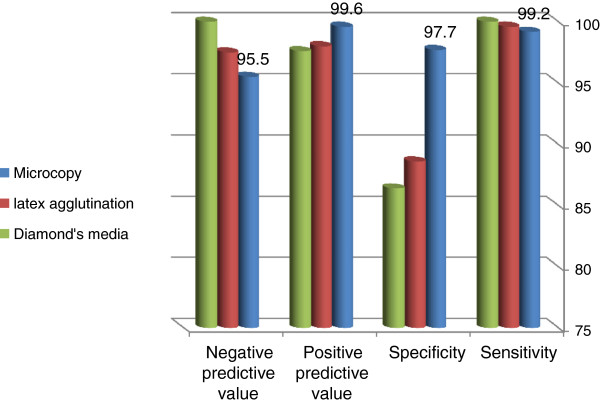
**Accuracy of microscopy, latex agglutination, and Diamond’s media compared with PCR for detecting *T. vaginalis.***

During the study period there were 542 admissions to the hospital. The majority of them (364, 67.2%) were obstetrical cases and the rest (178, 32.8%) were gynecological cases**.** There were 297 (35.1%) women with history suggestive of *T. vaginalis* and they were included in the study. There characteristics were shown in Table 1. Of the 297 women studied, 252 (84.8%) were positive for *T. vaginalis* by wet mount microscopy, 257 (86.5%) by latex agglutination, 253 (85.2%) by Diamond’s media, and 253 (85.2%) by PCR. The sensitivity and specificity of wet mount microscopy were 99.2% and 97.7%, respectively, compared with PCR. The sensitivity and specificity of the latex agglutination test and Diamond’s media were 99.6% and 88.6%, and 100.0% and 86.4%, respectively, compared with PCR (Table 2 and Figure 1).

**Table 1:** The mean (SD) of the basic characteristics of women enrolled to the study at Khartoum, Sudan

**The variable**	**Mean (SD)**
Age, years	28.7 (6.5)
Parity	3.4 (2.7)
Duration of symptoms, days	14.4 (8.3)
Body mass index, Kg/cm_2_	24.8 (3.4)

**Table 2:** **Accuracy of microscopy, latex agglutination, and diamond’s media compared with PCR for detecting *T. vaginalis***

		**PCR**			**Accuracy measure (95% CI)**	
		**Positive**	**Negative**	**Total**		
**Microcopy**	Positive	251	1	252	Sensitivity	99.2 (97.4-100.0)
	Negative	2	43	45	Specificity	97.7 (89.3-100.0)
	Total	253	44	297	Positive predictive value	99.6 (98.1-100.0)
					Negative predictive value	95.5 (86.1-99.1)
**Latex agglutination**	Positive	252	5	257	Sensitivity	99.6 (98.1-100.0)
	Negative	1	39	40	Specificity	88.6 (76.6-95.7)
	Total	253	44	297	Positive predictive value	98.0 (95.7-99.3)
					Negative predictive value	97.5 (88.3-100.0)
**Diamond’s media**	Positive	253	0	253	Sensitivity	100.0 (98.8-100.0)
	Negative	6	38	44	Specificity	86.4 (73.8-94.3)
	Total	259	38	297	Positive predictive value	97.6 (95.2-99.1)
					Negative predictive value	100 (92.4-100.0)

While wet mount microscopy had the highest positive predictive value (99.6%, confidence interval = 98.1–100.0%), Diamond’s media had the highest negative predictive value (100.0%, confidence interval = 92.4–100.0) in the diagnosis of *T. vaginalis* (Table 2).

## Discussion

The main findings of the current study were a high sensitivity and specificity of the investigated diagnostic tools (wet mount microscopy, latex agglutination test, and Diamond’s media). Additionally, wet mount microscopy had the highest positive predictive value and Diamond’s media had the highest negative predictive value in the diagnosis of *T. vaginalis*.

Recently, the sensitivity and specificity of wet mount microscopy were reported as 60% and 100%, respectively, whereas sensitivity and specificity of the culture (In-Pouch) system were 73.33% and 100%, respectively, when PCR was the gold standard in the diagnosis of *T. vaginalis*[15]. Moreover, Piperaki et al. [16] reported that PCR was the most sensitive method (100%), followed by culture (69.6%), wet mount (69.6%), and latex agglutination (54.6%) for detecting *T. vaginalis*. Likewise, Audo Sarkodie et al. [7] reported that the sensitivity of the latex agglutination test was 98.8% and specificity was 92.1% when an expanded gold standard based on the wet mount and culture results was used. In another study, Abraham and colleagues showed a high sensitivity (97.7%) [17], and Fernandez et al. showed a sensitivity of 86.7% and specificity of 95.1% for the latex agglutination test in detection of *Trichomonas vaginalis* in vaginal discharge [18]. However, inappropriate sensitivity and specificity (positive and negative predictive values were 6% and 100%, respectively) of the latex agglutination test were reported when culture was the gold standard [8].

One of the limitations of the current study was the selection criteria, where only symptomatic women were enrolled. This might be the reason behind the high rate of prevalence (approximately 85.0%) of *T. vaginalis* among these women, even different diagnostic tools were used. Another reason for the high rate of prevalence of *T. vaginalis* among the women in the current study might reflect the high sensitivity and specificity of the clinical diagnosis itself. Recently, a study showed that the sensitivity, specificity, and positive and negative predictive values of the clinical diagnosis compared with those of the culture method for *T. vaginalis* were 75%, 95%, 28%, and 99%, respectively [19]. Interestingly a high prevalence of (40%) trichomoniasis was reported among women in [20] Zambia, even among women who had never had sex [19].

## Conclusions

In this study, wet mount microscopy, the latex agglutination test, and Diamond’s media were found to be highly sensitive and specific. However, the availability and cost effectiveness might limit the use of Diamond’s media and PCR in routine practice.

## Competing interests

The authors declare that they have no competing interests.

## Authors’ contributions

AMS, GIG and IA designed the study. HSA, SMB and GIG conducted the clinical work. AMS and ABS conducted the laboratory work. IA, ABS and SMB performed the statistical analyses. All of the authors read and approved the final manuscript.
